# Giving Up the Guidelines: A Qualitative Evaluation of Disrupted Prescribing of Opioid Substitution Therapy in a Rural UK County During and Following the COVID-19 Pandemic

**DOI:** 10.3390/ijerph21121605

**Published:** 2024-11-30

**Authors:** Tim Lewington, Deanne Burch, Georges Petitjean

**Affiliations:** 1 Research & Innovation Department, Midlands Partnership University NHS Foundation Trust, St. George’s Hospital, Stafford ST16 3AG, UK; 2 Inclusion, Midlands Partnership University NHS Foundation Trust, St. George’s Hospital, Stafford ST16 3AG, UK; deanne.burch@mpft.nhs.uk (D.B.); georges.petitjean@mpft.nhs.uk (G.P.)

**Keywords:** qualitative, medical and non-medical prescribing, opioid substitution therapy, daily supervised consumption, COVID-19 pandemic, psychosocial therapy, telemedicine

## Abstract

The COVID-19 pandemic had wide impacts and repercussions for the NHS in the UK beyond the acute medical sector. This qualitative study evaluates the experience of medical (4) and non-medical prescribers (7) plus other staff (2 recovery workers; 2 community pharmacists) involved in opioid substitution therapy (OST) in a southern English county during and following the COVID-19 pandemic. Remote contact and a shift to predominantly weekly OST pick-up were anxiety-producing for clinicians, especially during the first lockdown. Widespread negative consequences were anticipated, such as a rise in fatal overdoses, which largely failed to materialise. Some diversion of medication was noted as were negative mental health consequences of enforced social isolation. Following a hiatus, psychosocial therapies transitioned to fully digital and subsequently hybrid delivery before returning to in-person group work towards the close of the pandemic. Changing power dynamics between clinicians and those accessing OST services were reported particularly around the re-introduction of daily supervised consumption and associated surveillance. Implications for future OST service delivery and national clinical guidance are suggested by way of conclusions.

## 1. Introduction

The COVID-19 global pandemic led to national restrictions on non-essential contact and travel in the UK beginning in late March 2020. Two major spikes in mortality followed—daily deaths of people with COVID-19 peaked in April 2020 and then again in January 2021 following a partial lifting of restrictions on social mixing in December 2020; a much lower third peak occurred in the autumn of 2021 as a booster vaccine was being deployed [1]. The pandemic triggered immediate and wide-ranging changes to clinical practice across the NHS [2]. Some services were suspended; other areas saw reduced service or adaptations to remote working and increased use of telemedicine [3]. In mid-April 2020, Public Health England (PHE) introduced draft prescribing advice and guidance for providers of services for people who use drugs and alcohol, acknowledging the importance of continuity of care for these groups and their coronavirus vulnerability.

Drug and Alcohol services in the UK are centrally funded by the Department of Health (DH) and were overseen by PHE with local funding distributed through county councils via a tendering process which produces a patchwork of public (NHS) and private local service provision (principally by third sector providers). People accessing services for opioid addiction are offered two complementary treatments; opioid substitution therapy and psychosocial therapy (PST). Both were severely disrupted by the pandemic. The *Drug Misuse and Dependence: UK Guidelines on Clinical Management* (also known as the Orange Book) provides clinical guidance for these treatments [4]. Whilst space limits our ability to review here the full 317 pages of guidance, a key element of OST is daily clinically supervised consumption (in practice, usually overseen by a pharmacist) during the early stage of treatment to allow safe titration of the medication and adherence to the regimen (reinforced by urine toxicology screening) and limit diversion. In effect, the Orange Book was suspended for a month during the first lockdown and only re-introduced in modified form for the rest of the first lockdown (national guidance was in effect replaced and supplemented with hastily assembled local guidance issued by service providers for roughly the first four months of the pandemic). In order to protect both those accessing services and staff from infection by COVID-19, key elements of treatment were all but suspended (daily supervised consumption, daily pickup, urine toxicology, and group psychosocial therapy were in abeyance, and face-to-face communication was replaced with remote access, principally by telephone during the early stages). There is little modern precedent for such a de facto suspension of official national clinical guidance at the same time as local service delivery was restructured wholesale, particularly when it concerns controlled substances.

A growing literature has examined pandemic changes to OST delivery internationally [5,6], and in various national contexts, particularly in North America [7,8,9,10]; Europe [11]; and Australasia [12,13]. In England, the policy context is shaped by the recent independent Review of Drugs conducted by Dame Carol Black [14], which informed a new 10-year government drug strategy, “*From Harm to Hope*” adopted in 2022 [15]; a critical review and resource for public health practitioners and policymakers followed [16]. An overview of relevant trends and an action plan have been developed by the successor to PHE, the Office for Health Improvement and Disparities [17]. Discussions of pandemic-affected service delivery in the devolved nations are available for Wales [18]; Scotland [19]; and Northern Ireland [20].

Croxford and colleagues [21] provide survey evidence for changing drug use by people who used drugs in England, Wales and Northern Ireland during the first lockdown of the pandemic, and found that 15% of people injecting drugs had increased the frequency of use by injection (with 85% staying stable or reducing) and an increase in cocaine injection compared to pre-pandemic; overall, smoking of drugs increased. Almost a quarter of their respondents reported changes to the composition of drug use (towards cocaine, amphetamines, pregabalin and other illicit substances), and a quarter reported greater use of alcohol. More than a third of respondents reported difficulty accessing health services and a quarter reported difficulty accessing sterile equipment. This is broadly consistent with other studies using qualitative methods which have examined the pandemic experiences of people accessing OST services in the Bristol area during the first summer of the pandemic [22]. Here, some changes were well received by those accessing services, especially less frequent OST collection and the lower incidence of supervised consumption. However, those accessing services were less enamoured with remote forms of communication and lack of face-to-face contact (findings broadly mirrored, despite different institutional contexts, in other studies internationally where regulation of OST was relaxed [7,13]).

A number of recent UK studies have begun to fill in the national picture of changes to service provision caused by the COVID-19 pandemic as well as the experience of those accessing these services. Carlisle and her colleagues [23] qualitatively investigate the recovery journeys of people in receipt of OAT (opioid agonist therapy) and highlight the influence of stigma in its multiple guises. London has also been the focus of a number of studies examining the changes to OST service delivery wrought by the pandemic [24]. Lambeth residents receiving OAT were positive about the more flexible approach to prescribing adopted early in the pandemic. Similar results were reported from the London boroughs of Haringey and Islington [25]. A wider study recruited a sample of service providers and people who inject drugs from across England and Scotland, which qualitatively examined the impacts of the pandemic and found support for the changes to more flexible prescribing, but scepticism amongst service providers that such changes would continue beyond the public health emergency [26]. Overall, the perspectives of those accessing services have been well covered but rather fewer studies have focussed on prescribing and service delivery in rural areas (see Scott and colleagues [27]). This evaluation contributes to the emerging picture of care and treatment for people accessing OST services in England during and following the pandemic, adding to the understanding of rural OST service delivery and the attitude of prescribers towards these changes. The significance of the study lies in building upon and reinforcing other accounts of pandemic-related changes to prescribing and their implications for the future provision of services (and the guidelines which frame clinical practice).

## 2. Methods

The original impetus for the study came from the two clinicians involved (GP and DB). The formulation of the research question reflected a qualitative focus: what was it like being a prescriber in a community drug and alcohol service during the first COVID-19 lockdown? The scope was subsequently expanded to cover the entire pandemic and its aftermath. The study design was an exploratory evaluation of prescribing practice under pandemic conditions.

Midlands Partnership University NHS Foundation Trust (MPFT) delivers drug and alcohol services across six counties in the UK; Hampshire was chosen as a focus for this evaluation as it covered a large and diverse area with a mix of urban communities and rural areas with a wide range of affluence and deprivation. The Hampshire service covers the predominantly rural areas of the county outside the two main cities of Portsmouth and Southampton (which were served by different providers). The service is delivered through clinical hubs located in 10 towns across the county.

A mix of medics and non-medical prescribers (NMPs) were interviewed remotely via Microsoft Teams (see Table 1) as were two recovery workers; all interviews were conducted by TL, a professional researcher with no prior connection to the clinical team. All the prescribing team were interviewed (bar one); recent hires and locums were excluded. Two local pharmacists, selected by convenience, were also interviewed in person in a private consulting space (though one did not consent to be recorded so contemporaneous handwritten notes were taken). An email invitation to interview was accompanied by an information sheet outlining the purpose of the evaluation (based on a staff list supplied by the central admin team). A semi-structured interview schedule, which was agreed by all authors, guided the interviews and verbal consent was recorded at the beginning of each interview. The transcripts were anonymised before the analysis. Interviews were mainly conducted in one sitting and ranged between 43 and 73 min in length (though the interviews with community pharmacists were shorter, at about 15 min).

Transcripts were coded independently by TL with the aid of Lumivero’s NVivo software (Version 13) and longhand by DB who subsequently met to discuss and agree on themes. A social constructivist approach guided the exploration of the data—that is, while we posit an objective, material reality, our interpretation of it is mediated through socially constructed theory and concept-laden discourse.

Interviews were all conducted in 2023. Recall of changes to clinical practice and allied events during the first lockdown was strong, though memories of the second and third lockdowns were less sharp, despite visual prompts and despite these events being more recent. This perhaps reflects the shock of sudden pandemic changes and the subsequent gradual increasing complexity of lockdown regulations in different areas and a more general pandemic fatigue during its second year.

The HRA decision tool confirmed that as an evaluation study external independent ethical review was not required (though the study was internally reviewed and placed on the Trust’s Evaluation Register with the identifier e2022-40).

The following findings are organised chronologically with major themes associated with each lockdown. These themes were as follows: differing interpretations of the Orange Book; clinician views on telemedicine vs. face-to-face contact; changing power dynamics; and clinician views of daily supervised consumption. The first lockdown is subdivided into two sections. We focus initially on the first 4 weeks, a period of great clinical uncertainty during which locally derived guidance superseded parts of the Orange Book. For presentational purposes, we have chosen a narrative format which highlights the chronology of events rather than themes, both for clarity of exposition and to contextualise themes in their relevant timeframe (for example, while the power dynamics between clinicians and those accessing services are important throughout and interwoven with other themes, such as the greater reliance on telemedicine, they are particularly relevant towards the end of the pandemic as attempts were made to restore daily supervised consumption).

## 3. Results

As the pandemic took hold in the UK and restrictions on social mobility were mooted in early 2020 it became apparent that OST would be severely disrupted, though the speed of the changes was not anticipated, as a senior medic noted [D2]: “*I guess no one quite anticipated the abruptness of the lockdown… I don’t think anyone knew what would happen”.* Table 2 provides a list of key dates with associated service changes.

### 3.1. Lockdown 1: The First Four Weeks

Once the lockdown began some staff were assigned to work from home and each hub began an urgent risk review of all OST prescriptions (clinic caseloads varied between approximately 200 and 400 OST prescriptions). The guidance codified in the Orange Book, while allowing wide scope for clinical discretion and flexibility, had been designed for routine clinical situations and were only partially suited to the task of dispensing in a pandemic; for example, on supervision:

“Levels of supervision should be based on an individual risk assessment for and with each patient… For most cases, it will be appropriate for new patients being prescribed methadone or buprenorphine to be required to take their daily doses under the direct supervision of a professional for a period of time to allow monitoring of progress and an ongoing risk assessment… In some cases, following this, the supervision will be needed for an extended period while for others it may be assessed as only being needed for a short period”.

(p. 101)

Clinical hubs were closed to all except one or two staff members at any one time; some pharmacies were on reduced hours or closed entirely; legal prohibitions restricted non-essential movement for the general population; consequently, all but a handful of those previously on daily supervised and daily pickup OST prescriptions were changed to weekly or fortnightly pickup without supervision. Internal guidance on OST collection assembled in the days before lockdown (dated 18th March) suggested those at the highest risk of non-compliance and/or overdose should collect in-person twice or three times weekly from pharmacies; people with lower risk could be assigned to weekly or fortnightly pickup—people accessing OST services with COVID-19 symptoms needing to isolate and who could not identify a buddy to pick up on their behalf were granted less frequent pickups or, in some cases, staff delivery. Naloxone was also supplied. In practice, clinicians opted for a preponderance of prescriptions with weekly pickup; later during the first lockdown a small number of rurally isolated, or people experiencing exceptional circumstances, received prescriptions with a duration of three weeks. The urgency of the task precluded consultation or joint decision-making with people prescribed OST. These risk assessments were guided by a combination of reviewing medical records; discussion with the regular prescriber and their recovery worker; and hastily arranged local guidance. Clinicians involved found the process enormously stressful:

*“It was quite nerve wracking, actually… So yes, but no, it was a very nervous time thinking, oh, my God, I’m giving all these people this amount of methadone”.*

[D3]

and

*“I had to go through their case notes, decide their risk and basically had to downgrade their risk essentially. Cross my fingers and hope for the best that they would be alright with a week’s worth of methadone or fortnight’s worth of methadone. As it stood, I only put about three patients on once a fortnight pick up”.*

[D1]

As the quote above indicates, despite reviewing a large number of people in a short space of time, risk was still individually assessed and minimised wherever possible within the constraints imposed by the pandemic. Nevertheless, clinical staff widely anticipated a variety of negative consequences, including but not limited to a rise in overdoses, with potentially fatal consequences: *“I struggled with it quite a lot really. I went home worrying, worrying terribly that they would take the whole two weeks. They’d overdose. They’d die”.* [N6]. Staff were also concerned about the clinical risk associated with COVID-19, particularly as some accessing services had poor general health and a sometimes distant relationship with primary care:

*“…are they going to contract COVID or are we going to have lots and lots more deaths not just because of drug use, but because of COVID itself? You know, some of our service users are really quite physically poorly”.*

[N3]

Rewritten prescriptions were transported to the various pharmacies used by each hub (itself a logistically difficult task due to the rural character of the county). Some nonmedical prescribers and recovery workers began daily welfare checks by phone in lieu of dosing supervision; psychosocial therapies were suspended until deep into the pandemic (discussed in more detail, below). Safeguarding concerns, particularly around children, were addressed by issuing lockable medicine boxes. Hampshire County Council began housing the homeless in a variety of temporary facilities. Early on, mobile phones were sourced and distributed to these new housing sites, allowing for communication with residents at regular, appointed intervals: *“we left one [phone] at the hostel, it meant that anybody at the hostel who didn’t have a phone, that was our phone to be able to contact them at an arranged time”* [N1].

In response to the high levels of staff anxiety, a variety of measures were quickly instituted. The county-wide team began daily prescribers’ meetings to review and discuss individual cases and to offer general support to each other. While prescribers remained individually responsible for their own prescriptions, daily discussions lessened anxiety, particularly around those individuals with more complex needs and considerations. A daily complex case review was also introduced, where prescribers could seek advice and reassurance on complicated cases.

The pandemic radically disrupted routine, upturning well-established and settled practices while creating fear, anxiety and uncertainty in its wake. Despite the effective suspension of the Orange Book (at least for the first month of the first lockdown, and in diluted and modified form for the following three months), minimising risk remained central to the reassessments of OST prescriptions. As the clinical lead observed, *“…at the time we didn’t do any titrations. Nobody was on reductions… Trying to keep everything as stable as it possibly could [be]”.* Additionally, various support mechanisms were created locally within Hampshire and more widely in the Trust, but the changes were profoundly destabilising nevertheless: *“… we had to do something. We couldn’t just say, you know, let’s shut the door till this is over”* [N6]; and, *“I think it was the fact that we’ve all worked so hard to get these qualifications and then it just felt like we were ripping up the book and doing our own thing. That was the thing that created the fear…”* [N2]. Both medics and NMPs sought reassurance and clarification from the medical lead about the legal status of their prescribing and the potential consequences should serious repercussions ensue (the reassurance came in the form of a *“… fairly standard reply: ‘Everyone understands, everyone’s in the same boat, everyone’s doing the same thing’”* [D2]).

### 3.2. Continuation of Lockdown 1: April to July 2020

PHE issued new interim, draft clinical guidance from mid-April 2020 directly to service providers. This limited guidance was regularly revised to accommodate the evolving pandemic, changes to national lockdown policy, and the safety and protection protocols surrounding COVID-19 (the guidance was eventually published in mid-July and remained in force for 12 months [29]). Even with the provision of extra emergency facilities in the shape of “Nightingale” hospitals [30], concern remained that the NHS may become overwhelmed by the scale of the pandemic. The new guidelines continued to emphasise the need to relieve pressure from acute services wherever possible and to protect those populations particularly vulnerable to COVID-19, such as people with drug or alcohol dependence. By July, specific guidance was introduced for a return to compliance with the Orange Book “[w]here safe to do so”; and “[a]rrangements for prescribing and dispensing of medicines used in drug and alcohol treatment were previously changed to take account of service and pharmacy closures, staff unavailability, patients having to maintain social distance or self-isolate (including the clinically extremely vulnerable), and the need to reduce the spread of COVID-19. These arrangements should continue to be reviewed” [29]. The new guidance continued to recommend buprenorphine for people new to services but those choosing methadone were recommended to pick up daily initially and take-home doses thereafter “when appropriate” (though this was heavily mediated by pharmacy capacity and capability locally). The guidance recommended keeping face-to-face interaction to a minimum while resuming treatments, such as detoxification, OST supervision, biological drug and BBV (blood-borne virus) testing using appropriate personal protective equipment (PPE). In reality, this began a process of restoring services whose speed and extent were dependent on the availability of local staff (in clinics and pharmacies) and other resources, and would result in a patchwork of service provision which varied locally and which ebbed and flowed over time.

Regular three-monthly, face-to-face prescriber reviews, suspended since the beginning of the lockdown, were reintroduced but in a controlled and highly choreographed manner; the focus was limited to initial assessments for those new to services (that some physically vulnerable staff still needed to be shielded by working from home provided another constraint on face-to-face contact): *“We were actually doing a proper assessment of the new patients. But even then, we were still doing telephone consultations on pretty much everyone else”* [D1]. Clinic hubs introduced a one-way system guided by yellow tape on the floor. A maximum of two staff members, wearing full PPE (personal protective equipment, including visors, masks, gowns and gloves) shepherded mask-wearing people accessing services to various stations where toxicology screening was completed (this stage usually guided by a recovery worker), followed by a socially distanced meeting with the prescriber in a separate room:

*“Someone would knock on the door. They were asked the questions, have they tested [for COVID-19]; have they symptoms? And then they could come in. And of course, we had to be kitted up and everything. And the floor was marked with tape (this is funny thinking back on it). And they would have to stand in a particular area. And they were given say the urine sample [containers]. Our set-up here is when you walk into reception, the toilets are right here… So they could go, do, go back to the waiting spot and then go and sit down in the vastly spaced out, temporary, an NMP room, if you like”.*

[R1]

To begin with, half-hour fallow periods between appointments were necessary and contact surfaces were regularly disinfected: “*… at one point I was mopping a floor in between seeing every patient… I distinctly remember that; a lot of wiping, a lot of mopping and a lot of plastic*” [N1]. Clearly, the time-consuming protocols surrounding face-to-face appointments limited their number and regulated the speed at which in-person services could resume. While those perceived to be at the highest risk were prioritised for in-person reviews, a much greater reliance on telemedicine ensued (supported by the large proportion of staff working from home). Nevertheless, various hurdles remained, not least widespread staff suspicion of the clinical efficacy of telephone consultations:

*“It wasn’t a suggestion, it was an order really, that we couldn’t see anybody. We could literally not have face-to-face contact with our clients and patients anymore. And yeah, so we had to get used to using the telephone…. But the idea of not seeing patients was really tough… And I found that incredibly difficult to get my head around. How you could offer a patient, an opiate treatment program without seeing them, without getting a drug test; I’ve spent 20-odd years relying on drug tests in order to prescribe treatments. It was very, very difficult to manage”.*

[D2]

and

*“I personally take the view that telemedicine is not really medicine. I mean, I’m quite old fashioned and I think that you need to eyeball your patients. I think there is a reason why doctors and nurses have been in the same room as their patients for centuries, and that’s because we can’t really know what’s wrong with them and we can’t really see how unwell they are unless we see them”.*

[D1]

While medics focused on the need to visually assess signs and symptoms of various health conditions (and not limited to those related to OST), a level of distrust was evident from some clinicians which went beyond the simple limits of telephone technology:

*“For me, during the telephone consultation, sometimes it was hard to trust anything that is said in the background because first of all, you don’t know who you are talking to, in that particular person. Number two, sometimes you will hear somebody in their background like, you know, like maybe coercing or telling them or say this or you don’t know really what’s going on”.*

[N6]

and

*“In some of it, you know, they could say what they like because I couldn’t see them and I couldn’t test them. So testing was really difficult. So I kind of felt that some people did quite well in lockdown and other people’s fabrication increased of what they were telling me”.*

[N4]

A few clinicians took a more positive view of telephone interaction, recognising that for some people accessing services there were benefits:

*“But sometimes if someone’s suffering chronic anxiety and I’m sure during COVID, if you already suffered with a mental health problem, it was going to make it a hundred times worse, but I found some, a group of individuals maybe that suffered from high anxiety or, you know, that just talking they were able to open up more because they weren’t in front of me. It was a phone call; on the phone they were a bit more relaxed about it”.*

[N6]

The few people new to services during the first lockdown became the focus of clinical anxiety as all initial assessments had to be completed by telephone, absent toxicology screening or daily supervised consumption (*“I think that was probably the scariest thing that we did“.* [D1]). There was particular concern around titration for new individuals:

*“…we would also have to start people, do titration, but be a bit more careful about the titration than perhaps we would do if they were supervised. So slower titration, you know, maybe taking a longer time to get them up on the doses in order to have fewer pickups at the pharmacy*.

*Although in a way, the Orange guidelines were out the window, but in an another way they weren’t, in that the principle of maintaining methadone tolerance is really important and we were trying to avoid the risk of people being dangerously re-titrated or coming off script and being at risk of overdose from heroin. So we felt that the evidence for people being on a script and being safer on a script than off script, even if that meant slightly less monitoring of that script, that was still the lesser of two evils, clinically”.*

[D1]

In the first few weeks of the lockdown, there were few new people to assess as prison releases were disrupted (court activity was suspended so people in prison on short-term drug-related remand, remained in prison for longer than usual)—there were perhaps only *“one or two a week maximum”* [D1]. Telephone assessment substituted for an in-person assessment and special measures were instituted for those few new to services who did present:

*“…so somebody would come to the front door, we’d give them a phone, they’d go off and sit somewhere and I’d ring them because we weren’t allowed to see anyone face-to-face, you know? So it’s a bit strange. They’d be in the car park over there and I’d be here and I could probably wave at them, but not actually see them”*.

[N1]

As the lockdown progressed and street heroin became scarcer, demand for initial assessments increased: “*the supply of heroin went down; you clearly weren’t meant to be out; so attempting to score heroin made you very visible. So for lots of reasons, people were wanting to get on treatment*” [N1]. Where the presentation was of someone previously known to the service, the clinical history was available to inform the prescribing decision. Completely new presentations were more problematic, however, due to the lack of objective evidence of opioid tolerance:

*“The harder cases were the ones that were previously not known to services, but were presenting as new heroin users. … We weren’t doing any urine screens initially, so for new patients, so every single new presentation was having to be discussed at a very high level with the kind of clinical risk team, you know, centrally at the Trust. And so we were having to do Teams meetings, present the patient, the history and then make a decision as to scripting, and then we would have to get the script to the pharmacy”.*

[D1]

From mid-July, while daily supervision was returned to the guidelines as an option, it was often restricted by local circumstances. A pharmacist testing positive for COVID-19 for example, required the people collecting OST prescriptions to be redistributed to other available pharmacies—a task often complicated by various bans and restrictions imposed on those thought to pose a high risk of theft. One pharmacist interviewed for this project, whose pharmacy counter was based at the rear of a large store in a commercial centre, struggled to separate those prescribed OST from others waiting at the counter, and had little or no control over those who entered or exited the mall-based store. Petty theft of sales items was a major concern for pharmacists and was managed by placing restrictions on the total number of people collecting OST prescriptions and an appointment system for receipt of medication and, later on, supervision (reported by both community pharmacists interviewed).

### 3.3. Brief Easing of Restrictions Followed by Lockdown 2 and 3: July 2020 to March 2021

From early summer, COVID-19 restrictions in England had begun to be eased; pubs and restaurants were permitted to re-open in July. The number of new COVID-19 infections and the death rate fell throughout the summer—the first licenced AstraZeneca vaccine would be introduced in the UK in January 2021 [31]. As infections started to increase again from mid-September 2020, restrictions were re-introduced, limiting both indoor and outdoor social gatherings. As the NHS came under increased pressure during the autumn, a second lockdown was instituted lasting from early November to early December. Permitting limited social mixing over the Christmas holiday period duly led to a spike in infections and deaths in January and a third and final lockdown was mandated (beginning on January 6th and lasting for two months, with restrictions gradually relaxed in stages over the following four months).

From November 2020, a meeting of all of the Inclusion Drug and Alcohol Treatment prescribers was instituted and met quarterly to share concerns and best practice. From these meetings a service-wide set of idealised scenarios were developed by the Quality Team (a central team focussed on ensuring high-quality services and governance) in an attempt to anticipate local conditions and circumstances and to address the lack of national guidelines:

*“The trust would regularly send out kind of notices that updated guidance on how to proceed with different scenarios. And I think we got to version 12 or 13, sometimes with quite minor revisions, but they would set out the parameters for what we should and shouldn’t, what we could and couldn’t do and how we would approach different situations. And that those guidance guidelines, I think, were helpful and reassuring as we were sort of making it up, we were making the plan up together rather than each hub maybe trying to do its own sort of way forward in this muddle”.*

[D4]

The clinical lead for Hampshire described the logic for the daily prescribers’ meeting:

*“I hoped it would make the prescribers feel that they weren’t working in isolation. That other people were actually experiencing some of the difficulties that they were coming up against. Or, you know, and then we could all make a decision collectively, all together. And if there was anything that we couldn’t deal with in that meeting, then that would then go to the Quality Team for discussion the following morning”.*

At the start of the pandemic, lockdown restrictions had shut down PST activities entirely: *“…they completely disappeared overnight”* [D2]. By the autumn, equipment (webcams and TVs) had been obtained to restart online-only group work, initially in a single, large group which operated across the county. Tablets and SIM cards were also sourced to allow those without access to join. While not universally welcomed, some individuals accessing drug and alcohol treatment favoured the convenience of this delivery mechanism; recovery workers, who reported the frequent reluctance to attend this type of group work in-person prior to the pandemic, found digital delivery to be more accessible and acceptable: *“… a lot of the services found it very positive because instead of having chronic anxiety and not wanting to step out the door and come to a group, they just clicked on the computer and they saw other people and they didn’t feel so anxious… [and] that the numbers grew with the online groups. You know, people that had never engaged in groups in their lives were coming”.* [N6].

The initial, single county-wide group proliferated and broke into smaller groups over time but were still only offered online, at least for the duration of the lockdowns. In consequence, clinics remained sparsely populated with no drop-in services offered or open access (doors remained locked for most of the time). Some clinicians preferred the relatively ordered, less spontaneous and quieter clinics which resulted: *“I don’t miss the open access. It’s pretty crazy at times. Very unpredictable”* [D2]. However, this negatively impacted the local culture of the clinic and had implications for how recovery-friendly the service was perceived to be:

*“So the whole atmosphere of the service changed from something that’s very inclusive and welcoming and this is your place and this is your community. To ‘no’ we are a service and you are out there and we are in here and we are protecting ourselves and we are protecting you and that I think is taking a long time to relax. We are having groups here now, but we’re not having open access and that’s partly we haven’t got staff to manage it, because we’re very thin on the ground…*

*And two years, three years down the line, we’re still not back to what we were. I don’t think we ever will be. I don’t like it. I don’t think that’s very recovery friendly”*.

[D1, interviewed in February 2023]

### 3.4. Post-COVID: Gradual Easing Towards a ‘New Normal’ from Spring, 2021

On an international scale, the World Health Organization declared the end of the global public health emergency in May of 2023 [32]. Pandemic-related social restrictions in England had been phased out by the late summer of 2021 as vaccine coverage rose. A ‘new normal’ would eventually emerge, incorporating many of the practices developed or adopted during the public health emergency. For OST services, the gradual return to pre-pandemic norms took 18 months or longer and included permanent changes to practice developed during the pandemic. These changes and continuities covered three principal areas: PST; telemedicine and prescribing. We will deal with each of these in turn.

#### 3.4.1. Psychosocial Therapy

Once equipment was sourced and restrictions relaxed allowing a small number of people to meet in a single room, recovery workers trialled hybrid group work. Depending on the configuration of the clinic, six or eight people could be accommodated with a similar number joining remotely. Those with a preference for physical attendance joined a rota to attend in person, while tablets were distributed to those lacking the means to join remotely:

*“So we had six people in the group room is what they decided and they’d have to be spaced out. And then we bought all this technology. So we managed to get a budget for to buy some decent cameras and a decent screen and we’d have people accessing it at home.*

*… you’d have to take a list of names of people that may want to come in next week and you’d have to rotate so everybody got an opportunity to come in”.*

[R1]

These hybrid groups proved more popular than in-person PST though they were phased out in favour of in-person-only group sessions by March 2022. In contrast to pre-pandemic, most clinics have maintained restricted access, allowing drop-in sessions only at certain limited times in favour of locked doors and appointment-only entry: “*…the open access never really started again*” [N3], though no-one in crisis is turned away.

#### 3.4.2. Telemedicine

Pre-pandemic, if someone accessing services failed to attend an appointment, be it a three-monthly prescribing review or monthly visit with their assigned recovery worker, consequences would automatically follow:

*“Whereas pre-COVID, if you don’t come in face-to-face, there’s going to be huge consequences. Whereas now, it’s like, it’s fine. As long as somebody is reviewed face-to-face, then the next one can be a telephone or Teams. And we’re okay about that now. You’ve seen your key worker”.*

[N6]

Some people accessing services (especially those living in less accessible, rural locations) found a greater reliance on telephone and video consultations to be both more convenient and encouraged greater engagement with the service:

*“So a lot of the non-engagement clients would answer their phones a lot. And all of a sudden they were answering their phones because… they felt a bit more respected rather than being treated like a child”.*

[R1]

Prescriptions withheld until a face-to-face visit or a return to daily supervised consumption had previously been perceived in punitive terms by those accessing services and these measures were now deployed less frequently by some clinicians, where evidence was available of on-going phone or video contact. As previously noted, clinicians tended to have strongly polarised interpretations of the value of telephone contact; *“Now we’re out of the pandemic, and I don’t use the phone unless I absolutely must. I didn’t find it valuable at all”.* [D2] and the convenience and increased engagement are not seen to outweigh the clinical benefits of face-to-face contact:

*“I really wish they’d come in. So that is a bit of a perhaps power dynamic struggle between patients saying, Oh, just ring me, just ring me. And I’m saying, No, I can’t ring you. I need to see you. ‘Why do you need to see me?’ You know, well, it’s important. Now we’ve developed contact that has a mix of the telephone and direct contact so that’s one of the things that changed from the pandemic that we are able to use telephone contact, but I don’t find it terribly helpful”.*

[D2]

Another medic found positive value in telemedicine but remained sceptical of its overall contribution:

*“I did enjoy doing some telephone reviews, and I found that we had some really good, nice chats down the phone; patients didn’t feel all that threatened; they knew they didn’t need to give a urine screen; we could just have a chat. And I think that there was some positivity to that… But I think on the whole, not seeing our patients is detrimental to their health and detrimental to their recovery because I think it’s stalled their recovery”.*

[D1]

The changing power dynamics between those accessing services and clinicians post-pandemic is evident in attempts to return to pre-pandemic levels of daily supervised consumption and other conditionality placed on prescriptions, explored below.

#### 3.4.3. OST

The expectation amongst all the clinicians interviewed was that a large increase in overdose deaths across the county would result from transferring a large number of prescriptions (internal figures indicated daily supervision comprised just over a third of the average monthly total OST prescriptions prior to the pandemic) from daily supervised consumption to mostly weekly pickup without supervision (with more frequent pickup for the highest risk group), but this proved to be largely unfounded:

*“I think we were all braced for drug-related deaths to go through the roof and them selling their methadone to each other and, you know, all these risks. And actually it didn’t happen”.*

[N6]

and

*“I don’t recall anything unusual happening. Despite my concerns about people being on a weekly, monthly, monthly in some cases, scripts… but in terms of deaths; we didn’t have any obvious harm come about as a result”.*

[D3]

Clinical concern was expressed about the inability to control risk associated with titration if someone shared their take-home supply with another person using opioids and some small-scale diversion was reported. Other risks were mitigated through the provision of naloxone, locked medicine boxes, restored needle exchange services and regular welfare checks by telephone.

The cessation of supervision and the shift to mainly weekly pickup impacted the relationship between clinicians and those prescribed OST which constituted both a shift in power dynamics and a ‘maturing’ of the relationship, with some clinicians becoming more inclined to treat people who use drugs with greater trust (and even people entirely new to services were effectively fast-tracked to a minimally intrusive prescription regimen). Clinicians noted the benefits of the new, more flexible relationship:

*“I think it helped them gain more control over when they can take their medication. You know, because a lot of people like to take their OST first thing in the morning because they’re uncomfortable. And if you’re supervised you can’t do that, you’ve got to wait to get to the chemist. And sometimes then by the time you wait to go to the chemist, you’re uncomfortable. You’re achy. So what do you do? Do you go to score or do you go to the chemist. So actually, it did really help people have a bit more control over the treatment”.*

[N1]

Also,

*“So the positive or maybe the learning outcome for me, say as a prescriber in that time was these service users didn’t mess around with it. They knew it was a valuable thing and a lot of them gave clean tests… It was a real positive that they didn’t have someone telling them what they had to do and not trusting them, if that’s the right word”.*

[N6]

and

*“I feel that giving them the two-week script was empowering… They had their own responsibility as to how they took it, as opposed to us imposing that you’ve got to go and take a set amount each day”.*

[N5]

Attempts to return to pre-pandemic levels of daily supervision, withholding prescriptions in response to missed appointments and so on, resulted in pushback from those accessing services (except those new to services who were unfamiliar with the more relaxed pandemic regime):

*“And that was the problem post-pandemic, because then, when you had to look at going back to working the previous way, you had really high-risk service users who had been on weekly pick-up who were then creating all sorts of problems when you said no, you have to go back onto more frequent pickup. So that created a problem for the relationship, because even if you discuss something in the clinical team meeting which you do, ultimately they see the prescribers that make the decision. So therefore their angst is against you. You can say 100 times, well, it’s a team decision. We discuss in the clinical team meeting and everyone has their say; they’re like, they know who signed the prescriptions. They know exactly, they come and see you. And if they want a change, it’s ultimately you that makes that decision”.*

[N2]

The exceptional efforts made by all concerned during the pandemic to keep people stable on OST prescriptions (going as far as clinicians delivering medication to people’s homes), combined with their demonstrably responsible use, suggested this more trusting relationship might endure:

*“I think there was also a sense in which the patients really picked up on the fact that we were going to get them their methadone by hook or by crook, we were going to get it to them. We were going to get the script to the pharmacy for them. We were going to deliver their methadone… We could move heaven and Earth to make sure they didn’t come off script. But there is a sense in which there’s been a power balance change, and maybe that’s a good thing, and I’m not saying it’s a bad thing”.*

[D1]

While the majority of the prescribers in the team thought on balance that the shift in the relations was a positive development, a minority were less sanguine and more inclined to distrust the testimony of those accessing services and to maintain rigid professional boundaries. However, some clinicians embraced the changes with enthusiasm; we close this section with the reflections of a non-medical prescriber:

*“It taught me that at that point in my prescribing career that sometimes… perhaps we hadn’t credited people with enough ability to manage themselves and manage their medications…*

*And I think giving people a little bit more trust and having, not holding everything so paternalistically, in a way, is, I think it’s been fantastic. I think that’s a really positive change. I think everybody, every prescriber works to the same guidelines. We have the same standard operating protocols. Those are a guide. And I think some people are more comfortable with… not deviating from them, it’s not the right word, but actually weighing up risk and saying, okay, we can try this because I feel that this is positive risk taking, and I think that’s something that COVID probably taught me, to do more positive risk taking, safely, of course, and not do mad stuff obviously. I think that’s got to be a good thing”.*

## 4. Discussion

The COVID-19 pandemic caused huge disruption across all sections of society; people were confined to their homes for extended periods; sections of the workforce were furloughed; school pupils and students were mostly taught remotely. Long-term consequences continue to be felt in both the private and public sectors as well as by individuals. Cancelled appointments, delayed diagnostics and courses of treatment during the pandemic exacerbated already lengthy NHS waiting lists which will take many years to reduce.

For drug services, the pandemic created a large-scale if entirely unplanned 18-month-long social experiment where national guidelines and local standard operating procedures, such as those in Hampshire associated with compliance and surveillance, were effectively suspended for a period and then subsequently re-introduced in modified form. A month-long emergency de facto suspension, during which all but a handful of prescriptions were re-prescribed to weekly or fortnightly pickup and where daily supervised consumption was not possible for almost all of the caseload, was followed by a three-month period where modified guidelines were applicable and only applied where resources allowed. Whilst the Orange Book was never formally withdrawn, and despite the introduction of various internal supportive measures, medical and non-medical prescribers found this to be a highly anxious period. Clinicians anticipated a wide range of negative consequences from the changes to prescription practices, particularly when combined with remote delivery of services and the cessation of psychosocial interventions. A partial list of expected impacts included a rise in fatal and non-fatal overdoses; increased diversion; safeguarding concerns; deteriorating mental health; as well as missed physical health complications from lack of direct clinical contact. For the most part, widespread fatal overdoses failed to materialise—though, nationally, there was some evidence of increased diversion and an increase in overdose deaths from methadone during the first lockdown [33] and some of the other negative consequences were successfully mitigated. Clinicians expected the lack of face-to-face contact, which was only gradually re-introduced as the pandemic eased, would hide emerging physical and mental health issues and this proved to be the case.

After the pandemic, while there had been no formal, service-wide attempt to engage in reflective practice or to share lessons learnt from the experience, individual clinicians had reassessed their own clinical practice and their relationships with people accessing services. Though radical departures from previous norms were not deemed necessary, an appetite for some measured and considered changes was apparent, especially in relation to positive risk-taking. This involves a reconsideration of risk and weighing of the balance of risk between restrictions imposed to promote safety (daily pickup and daily supervised consumption, for example) and the speed at which these restrictions are relaxed in order to promote self-management and ownership of recovery by those accessing services. The interplay between clinician’s perceptions and ability to manage risk, versus allowing people accessing drug services greater personal responsibility for managing their recovery (positive risk) suggests a potential focus for future research in this area. Some permanent changes to clinic procedures and prescriptions are nevertheless apparent; an increase in appointment-only visits and limited drop-in or open access facilities; fewer “script collect” prescriptions issued; and a slightly lower proportion of daily supervised consumption compared to pre-pandemic levels (internal Trust numbers show a drop of about 6%). While there are multiple factors which influence the proportion of the OST caseload receiving daily supervised consumption, it is likely the recent addition to the formulary of depot buprenorphine [34] and the ongoing cost pressures facing community pharmacists [35] will continue to constrain its use. This could be compounded by continued austerity as further budgetary retrenchment at the county council jeopardises services for the unhoused and others in a precarious social position [36]. Especially for those already socially isolated in rural areas, such as large parts of Hampshire, the ease of access to service hubs and associated public transport costs needs to be monitored to ensure future equity of access. The net impact of the combination of these positive and negative developments will require further investigation to determine.

Clinicians reported that people accessing drug services found many of the changes which introduced greater flexibility to be acceptable, and this reinforces evidence from elsewhere in the country and from other service providers [22,24,26]. Daily supervised consumption for initiates of OST and those deemed at high risk of overdose had been incorporated into clinical practice from 1996 and was incorporated into the 1999 edition of the Orange Book in a successful attempt to lower the growing incidence of fatal methadone overdoses [37]. Whilst daily supervision reduced the incidence of fatal methadone overdoses, it remained unpopular with those accessing services in the lead-up to the pandemic [24]. Longer-duration pickups during the pandemic allowed recipients greater control and flexibility over the timing of dosing and their daily activities. Clinicians recognised the potential empowering benefits of demonstrating a greater degree of trust implied in reduced surveillance. Perhaps the hardest aspect of service delivery to evaluate is the role of a local team or hub ‘culture’—in this case the intangibles associated with open access, friendliness and inclusion. The idea of either excluding or social distancing those seeking treatment runs counter to the ethos and practice of clinicians, and while some of the difficulties associated with managing open-access clinics in Hampshire will probably not be reproduced post-pandemic, a new balance between face-to-face contact and greater use of telemedicine will likely reflect the wider emphasis on digital delivery across the NHS.

Following the first and most acute phase of the pandemic, a period of fluctuating social restrictions and corresponding phased restoration of services led to a partial return to pre-pandemic norms. Remote, group-based psychosocial therapy was re-introduced following a hiatus, as were face-to-face prescription reviews (albeit at six-monthly intervals in the first instance). Daily supervised consumption was re-introduced, where local pharmacy capacity allowed. These changes led to pushback from those accessing services and an attempt to modify the power dynamics present (including maintaining the convenience associated with telemedicine, negotiating down the consequences of missed appointments and resisting attempts to re-introduce supervised consumption). While overall the reaction of Hampshire clinicians to pandemic-related changes was mixed and they remained acutely aware of the continued risks associated with OST, clinicians in this study welcomed many of the service changes (including questioning the proportion needing to be prescribed daily supervised consumption and being more open to a speedier transition towards a pickup based regimen). Whilst the study sought to gather the clinician experience within one service, these findings are broadly consistent with those from elsewhere in the UK [22,24,26] and we concur with the findings of Tom May and his colleagues that “… providers became more comfortable with flexible dosing and agreed that future discussions regarding OST were warranted, given how limited incidents of harm were reported” [26] (p. 6).

### Strengths and Limitations

This study adds to the emerging national picture of the changes to OST prescribing in England during the pandemic and clinicians’ attitudes towards them. Clinicians reflected on their loss of control over key aspects of both prescribing and wider treatment during the pandemic and how the power dynamics with those accessing services impacted on the restoration of treatment services. The principal limitation of the study is the lack of input from those accessing services. A further limitation could be the potential for recall impairment as events across the previous two years were reviewed, though as previously noted, recall of the first lockdown was more detailed than subsequent lockdowns and changes in national COVID-19 policy. While acknowledging the possibility, it is hoped that any negative impact of internal power relations impacting perceptions and interpretations was mitigated by interviews being conducted by a researcher with no previous contact with the clinical team in Hampshire.

## 5. Conclusions

The long tail of the pandemic continues to impact drug treatment and other NHS services. Open access to clinics and a welcoming, inclusive local culture for those accessing services in Hampshire has yet to be fully restored for example. In the context of OST prescribing, slightly lower numbers of daily pickup and daily supervised consumption suggest Hampshire prescribers have a greater propensity for positive risk-taking by more speedily transferring those on daily supervision to less frequent pickup of medication without supervised consumption. Though clinicians had mixed views on the prescription changes wrought by the pandemic, a majority felt there was scope for a less intrusive supervision and surveillance regimen, which may have positive impacts on the relationship with those accessing services.

## Figures and Tables

**Table 1:** Hampshire MPFT OST staff interviewed.

Identified in Text as:	Staff Role	Staff Characteristics
D1 to D4	Medical	2 psychiatrists, 1 GP, 1 hospital doctor: 5 to 18 years of experience
N1 to N7	Non-medical nurse prescribers	4 to 11 years of experience
R1 and R2	Recovery workers	9 and 4 years of experience

**Table 2:** Key dates in the COVID-19 pandemic and associated OST service changes.

Key Dates:	COVID-19 Restrictions:	OST Service Changes:
First Lockdown: 23 March 2020 to 4 July 2020	Only essential workers permitted to travel. Trips outside the home limited to food and medical provision. Restrictions eased from June (schools/non-essential shops opened).	Supervised consumption and daily pickup halted. Replaced with mostly 1- and 2-week pickup. No supervision. No face-to-face contact with people accessing services. Telephone welfare checks.
Partial Easing July 2020 to October 2020	Non-essential retail re-opens from mid’ June. Pubs, restaurants, hairdressers re-open. Indoor ‘rule of six’ introduced in September. Restrictions re-introduced in October.	Face-to-face for initial assessments followed by resumption for some 3-month reviews. Remote digital Psychosocial therapy (PST). Re-introduction of some supervised consumption. Phase-out of fortnightly pickup (beginning with those at high risk). Online-only PST introduced
Second Lockdown 5 November 2020 to 2 December 2020	Lockdown plus support “bubble” instituted (where a vulnerable household could link with 1 other household for support).^1^ 1 person outside could be met outside bubble.	Face-to-face assessments and reviews curtailed. Return to telephone consultations supplemented with video consultations.
Third Lockdown 6 January 2021 to 8 March 2021	Stay at home ordered (with a few exceptions) due to Alpha variant.	Continued reliance on digital communications with people accessing drug treatment services.
Easing March 2021 to July 2021	March to July various step-downs.	Hybrid PST introduced.

^1^ Further information on ‘support bubbles’ can be found here: https://www.gov.uk/guidance/making-a-support-bubble-with-another-household. (accessed on 5 August 2024). SOURCE: Adapted from Institute for Government, (2022). *Timeline of UK Government Coronavirus Lockdowns and Measures, March 2020 to December 2021* [28].

## Data Availability

The data presented in this study are available on request from the corresponding author. The data are not publicly available due to privacy restrictions to protect the anonymity of interviewees.
